# Clinical, Biological and Genetic Analysis of 8 Cases of Congenital Isolated Adrenocorticotrophic Hormone (ACTH) Deficiency

**DOI:** 10.1371/journal.pone.0026516

**Published:** 2011-10-18

**Authors:** Luu-Ly Pham, Christelle Garot, Thierry Brue, Raja Brauner

**Affiliations:** 1 Université Paris Descartes, Sorbonne Paris Cité, and Assistance Publique-Hôpitaux de Paris, Hôpital Bicêtre, Unité d'Endocrinologie Pédiatrique, Le Kremlin Bicêtre, France; 2 Centre de Référence des Maladies Rares d'origine Hypophysaire, Centre Hospitalier Universitaire Timone, Assistance Publique-Hôpitaux de Marseille and Université de la Méditerranée, Marseille, France; Hôpital Robert Debré, France

## Abstract

**Background:**

Congenital isolated adrenocorticotrophic hormone (ACTH) deficiency may be rare, but it could be an underestimated cause of neonatal death. Our objective was to shorten the time between first symptoms and diagnosis.

**Methods:**

This single-centre retrospective case-cohort study was carried out on eight consecutive patients.

**Results:**

Two had the neonatal form and 6 the late onset form. Six were admitted to an intensive care unit at least once for seizures with hypoglycemia, major hypothermia, fever, and/or collapsus. The 2 neonatal cases presented with hypoglycemia and in a state of “apparent death” at birth or hypothermia (29°C) at 6 days. All 6 late onset cases had also been admitted to an emergency department 1–3 times, but had left hospital incorrectly diagnosed. Their first symptoms were noted at 3–12.3 years, and they were diagnosed at 3.3–14.4 years. All had hypoglycemia, and 4 had had seizures. The presenting symptoms were vomiting and/or abdominal pain, asthenia, irritability, difficulty with physical activities, and anorexia. The school performance of 4 deteriorated. Two underwent psychotherapy and treatment for depression, which was stopped when Hydrocortisone® replacement therapy began.

The plasma concentrations in spontaneous hypoglycemia were: ACTH<5 to 17.1 pg/mL, with concomitant cortisol <3.5 to 37 ng/mL. The plasma dehydroepiandrosterone sulfate (DHAS) concentrations were low in the 7 evaluated. The coding sequence of TPIT was normal in all.

**Conclusion:**

Several unexplained symptoms in a child, mainly gastro-intestinal symptoms and seizures due to hypoglycemia, may indicate ACTH deficiency. A low or normal basal plasma ACTH despite concomitant low cortisol at 8 a.m. and/or in spontaneous hypoglycemia, associated with low DHAS, in a patient not given corticosteroids is highly suggestive of ACTH deficiency. The isolated character of ACTH deficiency must be confirmed by determining the other hypothalamic-pituitary functions, and Hydrocortisone® replacement therapy initiated in emergency.

## Introduction

Congenital isolated adrenocorticotrophic hormone (ACTH) deficiency is a rare condition that was first reported in 1954 [1]. Its prevalence is unknown, but it is considered to be an underestimated cause of neonatal death [2]. It is difficult to diagnose because its symptoms are various and unspecific, and its biological expression difficult to interpret, particularly in neonates, as they have physiologically low plasma cortisol concentrations. The patients may have no symptoms of the disease until decompensation occurs, which may be fatal.

There are two forms of isolated ACTH deficiency in childhood. The early onset form appears before the age of one or two years and the late onset form after 3 years. To our knowledge, 42 cases of children with isolated ACTH deficiency have been reported. Vallette-Kasic et al [2] reported 27 neonatal cases having ten different mutations in the TBX19 (TPIT gene) in 17 patients. Metherell et al [3] reported 7 cases, 4 of whom were less than one year old, with two different mutations in 2 of them, and 3 of whom were over 5 years old. Nozue et al [4] reported one Japanese case and collected 7 more cases, they included 3 early onset and 5 late onset forms. Mutations in the TPIT gene are associated only with the early onset form [2]. A total of 12 loss-of-function TPIT gene mutations have been identified to date [5] with an autosomal recessive transmission pattern.

Objective: To shorten the time between appearence of the first symptoms and diagnosis by describing the clinical-biological presentation and the steps before diagnosis, and by defining the biological diagnostic tools.

## Materials and Methods

### Ethics statement

Written informed consent for the evaluations and for molecular analysis were obtained from the children's parents and included in the children's hospital medical record. All clinical investigations were conducted according to the principals expressed in the Helsinki Declaration. The Ethical Review Committee (Comité de Protection des Personnes, Ile de France III) stated that “this research was found to conform to generally accepted scientific principles and research ethical standards and to be in conformity with the laws and regulations of the country in which the research experiment was performed”.

### Patients

This single-centre retrospective case-cohort study was carried out on eight consecutive patients seen for congenital isolated ACTH deficiency by a senior pediatric endocrinologist (R Brauner) from 1990 to 2010. Diagnosis was based on low plasma basal concentrations of ACTH (normal 10–50 pg/mL) in spontaneous hypoglycemia and/or at 8 a.m. despite concomitant low cortisol (below 40 ng/mL or 110 nmol/L in the newborns; below 80 ng/mL or 220 nmol/L in the older children). No patient had been given corticosteroids.

### Methods

The history of each patient, including origin, consanguinity, familial diseases or unexplained death was recorded. Pre- and perinatal histories were reviewed for multiparity, gestational age, delivery, and perinatal abnormalities, particularly hypoglycemia, hypothermia and/or prolonged jaundice. The ages and causes of previous admissions to emergency departments were obtained from the parents and the children's health records, as were the statural growth curves, the weight changes during the year preceding the diagnosis of ACTH deficiency, and the school performances. Blood pressure and cardiac frequency were also recorded.

The plasma concentrations of growth hormone (GH) were measured during stimulation test (by arginine in case 1, ornithine in cases 5 and 7 or GH releasing hormone in case 8, Table 1), excluding the insulin test, or in spontaneous hypoglycaemia (in cases 2 and 4). GH was not evaluated in cases 3 and 6 because their growth rates and basal plasma concentrations of insulin-like growth factor 1 (IGF 1) were normal. A normal plasma GH concentration is above 10 ng/mL or 20 mIU/L. The other hypothalamic-pituitary functions were evaluated by measuring the basal plasma concentrations of thyroid stimulating hormone, free thyroxin and prolactin. The hypothalamic-pituitary morphology was evaluated by magnetic resonance imaging (MRI). The basal plasma concentrations of dehydroepiandrosterone sulfate (DHAS) were measured in all but case 2 and compared to the normal values for age and pubertal stage [6]. ACTH tests (250 µg) were performed on two patients (cases 4 and 8) by physicians from other teams during the follow-up and 12 h after Hydrocortisone® treatment.

**Table 1:** Characteristics of 8 cases with congenital isolated ACTH deficiency.

FORM	NEONATAL		LATE ONSET					
Case	1	2	3	4	5	6	7	8
Sex	F	F	F	M	M	M	M	M
Origin	France	France	France	France	Morocco	France/Colombia	Portugal	Algeria
Age at onset	Birth	6 days	3 yr	5 yr	3 yr	4 yr	9 yr	12.3 yr
Age at diagnosis	6 days	14 days	3.3 yr	5.3 yr	6.1 yr	8.8 yr	12.7 yr	14.4 yr
Diagnosis delay	6 days	8 days	0.3 yr	0.3 yr	3.1 yr	4.8 yr	3.7 yr	2.1 yr
Initial symptoms	Apparently dead	Hypothermia	Vomiting	Seizures	Seizures	Seizures	Vomiting	Seizures
Weight change yr-1, kg			−0,5	−2	NA	0	0	−1
N° admissions<diagnosis	1	1	2	2	numerous	3	1	3
Cardiac frequency, /min	Apparently dead	94	122	112	NA	120	NA	NA
Blood pressure, mm Hg	NA	51/24	104/59	85/45	100/60	95/45	80/50	low
Vomiting	0	0	+	+	0	0	+	+
Abdominal pain	0	0	+	+	+	+	0	+
Asthenia	0	0	+	+	+	+	+	+
School difficulties	0	0	0	0	+	+	+	+
Depressive or psychiatric symptoms	0	0	0	+	0	0	0	+
ACTH, pg/mL	4	11	<5	11.3	2	13	17.1	<5
Cortisol, ng/mL	20	12	30	31	<3.5	<10	37	3.5
DHAS, ng/mL	<60	NA	63	<30	<15	<15	160	112
Renin activity, pg/mL	39	NA	43	43	39	33	33	6
Aldosterone, ng/mL	56	NA	NA	36	NA	48.6	26	<2.5
Sodium, mmol/L	NA	139	133	126	NA	134	NA	NA
Potassium, mmol/L	NA	6	3.5	3.6	NA	4.4	NA	NA
Glucose, mmol/L	2.5	2.8	3.4	1.1	3.0	<1.1	NA	2.6
GH peak	22 ng/mL	28.5 mIU/L	NA	37 mIU/L	15 ng/mL	NA	9.9 mIU/L	18 ng/mL
IGF 1, ng/mL	47	77	55	59	219	123	249	100
Thyroid stimulating hormone, mIU/L	NA	6,6	2,1	0,45	NA	4,3	5,14	3,85
Free thyroxin, pmol/L	14	25	13,4	12,9	18	13	18,4	NA
Prolactin, ng/mL	32.5	20.2	NA	1,1	6	8.5	22	7.3
Age at last evaluation, yr	13	5	4.5	5.5	16.3	13.2	19.1	26.1

The plasma ACTH and cortisol concentrations at 8 a.m. of the patients' siblings were routinely evaluated, except for case 5.

Molecular analysis for mutations in the open reading frame of the TPIT gene was performed as previously reported [2]. DNA was extracted from peripheral lymphocytes and PCR-amplified using eight sets of flanking intronic primers for direct sequencing of exons.

## Results

Two patients had the neonatal form and 6 the late onset form (Table 1). All but cases 3 and 7 had been admitted to an intensive care unit at least once for seizures with hypoglycemia, major hypothermia, fever, and/or collapsus. All 6 late onset cases had also been admitted to an emergency department 1–3 times but had left hospital incorrectly diagnosed; they were diagnosed as suffering from encephalitis, gastroenteritis, asthma or faintness.

### Antecedents

We found no consanguinity, except for the parents of the maternal grandmother of case 2, who are cousins, and no familial hypothalamic-pituitary deficiency. The sibling of the maternal great grandmother of case 2 died soon after birth. The plasma ACTH and cortisol concentrations at 8 a.m. of the patients' siblings were normal.

All the patients were born at term (37 to 40 weeks of amenorrhea), two are non-identical twins (cases 2 and 7). The birth presentation was cephalic in 5 cases, breech in case 2 and unknown in two cases. The birth measurements were normal for 4 cases, below the 3^rd^ percentile for case 2, over the 95^th^ percentile for case 7 and unknown for case 5.

### Neonatal forms

The pregnancy for case 1 ended with pre-eclampsia, leading to interruption with the child in a state of “apparent death” and needing intensive care. Early, persistent hypoglycemia led to diagnosis and Hydrocortisone® replacement therapy. She is now aged 13 and in a good health.

Case 2 had difficulty feeding, oedema, and cold extremities at 6 days old; she was admitted to intensive care at 14 days with hypothermia (29°C). She was given Hydrocortisone® replacement therapy and is now aged 5 and in a good health.

### Late onset forms

No patient showed any features suggesting hypoglycemia, hypothermia or prolonged jaundice during the first 3 years of life. Two patients had been operated on (hydrocele in case 4 and amygdalectomy and appendectomy in case 7) without decompensation.

The first symptoms were noted at 3–12.3 years. They were diagnosed at 3.3–14.4 years, for a time between symptoms and diagnosis of 0.3 to 4.8 years.

The weight changes during the year before diagnosis ranged from a loss of 2 kg to no increase. All had hypoglycemia and 4 had had seizures. The presenting symptoms were vomiting and/or abdominal pain, asthenia, irritability, difficulty with physical activities, and anorexia. The school performance of 4 deteriorated, particularly their memory. Two (cases 4 and 8) underwent psychotherapy and treatment for depression, which was stopped when Hydrocortisone® replacement therapy began.

Case 6 had recurrent fever crises, leading to the diagnosis of Marshall's syndrome and corticosteroid treatment followed by a rapid improvement.

### Biological diagnosis, MRI data and genetic analysis

The plasma concentrations in spontaneous hypoglycemia were: ACTH<5 to 17.1 pg/mL, with concomitant cortisol <3.5 to 37 ng/mL (<9.7 to 102 nmol/L, Table 1). The plasma DHAS concentrations were low in the 7 cases evaluated. Plasma sodium was low in 3 of the 4 cases evaluated, with normal plasma potassium, normal plasma renin activity, and normal or low aldosterone concencentrations. Case 4 had a transient increase in transaminases during acute adrenal failure.

ACTH tests performed elsewhere showed no significant increases in the plasma concentrations of cortisol between basal and 60 min (from <3 to 3 ng/mL in case 4 and from 6 to 18 ng/mL in case 8).

The GH peaks were normal, except in case 7. The plasma IGF 1 concentrations were normal in all cases. The hypothalamic-pituitary morphologies evaluated on MRI were normal, except for a small (2 mm) anterior pituitary height in case 7.

The coding sequence of TPIT was normal in all.

### Evolution

The Hydrocortisone® replacement dose was adjusted on the basis of clinical well being, as the biological measurements were not helpful. It was 12 to 14 mg/m^2^/day, with careful and repeated information supplied to the parents and patients on the need to increase this dose in case of specific events, and to replace it with injections if there were any gastrointestinal symptoms or anaesthesia.

The symptoms disappeared and their school performances became normal. The scholastic development of all the patients was normal. Case 6 underwent central precocious, rapidly evolving puberty (onset at 9 years) despite the absence of any familial factor.

The growth rates of in all patients were normal. Three (cases 5, 7 and 8) reached their adult height (172, 180 and 171.5 cm respectively), which was similar to their target height.

## Discussion

The patients were of different origins and ages at diagnosis, there was no consanguinity or obesity. One underwent central precocious, rapidly evolving puberty.

### Diagnostic difficulties

Isolated ACTH deficiency may be diagnosed before birth. Weintrob et al [7] reported such a diagnosis *in utero* in a case caused by a new mutation in the TPIT gene. It was diagnosed based on a low estriol concentration in a second son after the first boy had died at 7 weeks due to a presumed cardiopathy. The mother's low estriol concentration was ignored because the fetal ultrasound was normal. They recommend that a low plasma estriol concentration in the context of a normal fetal ultrasound and growth, after excluding a placental sulfatase deficiency and Smith-Lemli-Opitz, should suggest deficient fetal steroidogenesis, with decreased adrenal production of DHAS.

The presenting symptom in neonates is frequently hypoglycemia. A normal or low plasma ACTH concentration excludes a peripheral adrenal deficiency, mainly congenital adrenal hyperplasia. Hypoglycemia is suggestive of this diagnosis if it is associated with jaundice and/or low blood pressure, but it may be isolated. Profound persistent hypoglycemia is a clear warning if it is not explained by neonatal conditions. As hypoglycemia is the best stimulus of ACTH and cortisol secretions, their plasma concentrations should be measured repeatedly in cases of spontaneous hypoglycemia, together with measurements of the other hypothalamic-pituitary hormones to determine whether the low ACTH is isolated or associated with other deficiencies. When interpreting the data, it is important to remember that the plasma thyroxin and GH concentrations are physiologically high in newborns. The concomitant insulin concentration should also be evaluated, to exclude hyperinsulinism as a cause of hypoglycemia. Only one case had a low GH peak associated with a small anterior pituitary height, but with normal plasma IGF 1 concentration at 12.7 years before puberty, followed by adult height at 180 cm, similar to his target height. We believe that this low GH peak was due to a transient prepubertal GH deficiency [8] rather than to the severe cortisol deficiency, as suggested by McEachern et al [9]. Congenital disorders of glycosylation type Ia (CDG syndrome) may also give rise to severe neonatal hypoglycemia due to the metabolic disorder, combined in certain cases with a cortisol deficiency (personal data). MRI can be used to exclude pituitary stalk interruption syndrome, which is the marker of a congenital hypothalamic-pituitary deficiency including GH deficiency [10]. In this situation, the association of GH and ACTH deficiencies may lead to severe hypoglycemia.

The great difficulty is that the plasma concentrations of cortisol are physiologically low in neonates. In doubtful neonatal situations, including unexplained hypoglycemia, and in case of low plasma concentrations of ACTH and cortisol despite the hypoglycemia, we give Hydrocortisone® per os (1 mg three times a day) and evaluate its effect on the blood glucose, and reassess ACTH and cortisol at 8 a.m. later.

Two of the patients seen for late onset form had been treated for depression. Almost all patients had had difficulty at school that disappeared once they began their Hydrocortisone® replacement therapy. Nozue et al [4] reported the case of a patient with isolated ACTH deficiency and psychomotor retardation, suggesting the specific role of the hypoglycemia and/or low blood pressure. Shulman et al [11] also reported symptoms that may mimic gastrointestinal illness or a psychiatric disorder, particularly behaviour changes or depression.

### Biological diagnosis and how to obtain a more rapid diagnosis

The vital prognosis is engaged by the ACTH deficiency, but diagnosis is difficult and sometimes missed: 6 of our 8 patients had previously been admitted to intensive care units at least once, and all had also been admitted to an emergency department. As the plasma ACTH is low, the symptoms suggesting a peripheral adrenal deficiency associated with increased ACTH (melanodermia and salt wasting) are absent. The plasma concentrations of ACTH and cortisol of all patients with unexplained hypoglycemia and/or non-febrile seizures should be measured during the hypoglycemia or soon after, if possible at 8 a.m.. We also recommend assaying the other hypothalamic-pituitary hormones before the patient leaves hospital. The biological diagnosis is based on a low plasma ACTH concentration despite low cortisol in a patient not given corticosteroids: an 8 a.m. cortisol lower than 40 ng/mL (or 110 nmol/L) suggests the diagnosis, while a value greater than 180 ng/mL (or 500 nmol/L) essentially eliminates it. Hyponatremia may also occur with the ACTH deficiency because of water retention due to a lack of cortisol to antagonize the effects of vasopressin [11].

We use the basal plasma concentrations of cortisol at 8 00 a.m. rather than ACTH-stimulated cortisol. Nasrallah et al [12] reported that some patients continue to have normal ACTH-stimulated cortisol concentrations despite being adrenally insufficient and suggest using the basal plasma DHAS concentrations. Almost all DHAS, the most abundant steroid in the circulation, is secreted by the adrenal glands, with a minor contribution by the testis. DHAS has a half-life of 10–20 h and does not follow a circadian rhythm. Thus a single measurement of DHAS is practical and reliable. These authors suggest that a normal DHAS concentration makes an ACTH deficiency extremely unlikely. All seven patients who we evaluated had a low DHAS concentration. However, the plasma DHAS is physiologically low during the first 5 years of life.

### Genetic reported data

Mutations of the TPIT gene are associated only with the early onset form [2], [3]. Vallette-Kasic [2] found 10 different mutations of the TPIT gene in 17/27 patients. All those with a mutation and only one patient without one suffered from hypoglycemia; the two groups differed in the frequency of cholestatic jaundice associated with hepatomegaly in 4 cases which disappeared after Hydrocortisone® therapy (11/17 of those with a mutation and 1/10 without), mean ACTH (6.8 pg/mL with and 12.1 pg/mL without) and consanguinity (5/13 with and 1/8 without).

Bremer et al [13] reported a case of isolated ACTH deficiency that presented as an acute neurologic emergency in a normal peripubertal girl with a normal TPIT gene.

Mutations in the gene encoding pro-opiomelanocortine (POMC) have been reported in patients with severe early-onset obesity, adrenal insufficiency and red hair pigmentation [14]. Mutation in the human encoding prohormone convertase 1 were found in an obese patient with ACTH and gonadotropin deficiencies [15]. Other candidate genes are those encoding corticotrophic releasing hormone and its receptor, but not alterations in these genes have been reported. This demonstrates both the variable clinical presentation and the need for continued investigation into the molecular mechanisms.

### Conclusion

This study has diagnostic implications. It is easy to measure blood glucose at 8 a.m. and to interpret the plasma concentrations of ACTH, cortisol, and DHAS in a child with symptoms such as gastro-intestinal symptoms, weight loss, scholastic difficulties and hypoglycemia, provided the patient has not been given corticosteroids. A low or normal basal plasma ACTH concentration, despite a concomitant low cortisol at 8 a.m. and/or in spontaneous hypoglycemia, associated with low DHAS, is highly suggestive of ACTH deficiency. No ACTH stimulation test is needed. The isolated character of ACTH deficiency must be confirmed by determining the other hypothalamic-pituitary functions, and Hydrocortisone® replacement therapy initiated in emergency.

The limitation of the study is the small number of the patients, explained by the rarity and the undiagnosed character of the isolated ACTH deficiency. We hope we have helped make physicians, mainly neonatologists and those working in emergency departments, more aware of this disease.

## References

[1] Steinberg A, Shechter FR, Segal HI (1954). True pituitary Addison's disease-A pituitary unitropic deficiency (fifteen-year follow-up).. J Clin Endocrinol Metab.

[2] Vallette-Kasic S, Brue T, Pulichino A-M, Gueydan M, Barlier A (2005). Congenital isolated adrenocorticotropin deficiency: an underestimated cause of neonatal death, explained by TPIT gene mutations.. J Clin Endocrinol Metab.

[3] Metherell LA, Savage MO, Dattani M, Walker J, Clayton PE (2004). TPIT mutations are associated with early-onset, but not late-onset isolated ACTH deficiency.. Eur J Endocrinol.

[4] Nozue H, Kamoda T, Imai H, Aoki T, Ichikawa K (2007). Isolated adrenocorticotropic hormone deficiency presenting with psychomotor retardation.. Pediatric International.

[5] Vallette-Kasic S, Couture C, Balsalobre A, Metherell L, Dattani M (2007). The TPIT gene mutation M86R associated with isolated adrenocorticotropin deficiency interferes with protein: protein interactions.. J Clin Endocrinol Metab.

[6] Forest MG (1992). Adrenal function tests. In: Ranke M (ed) Functional endocrinologic diagnostics in children and adolescents..

[7] Weintrob N, Drouin J, Vallette-Kasic S, Taub E, Marom D (2006). Low estriol levels in the maternal triple-marker screen as a predictor of isolated adrenocorticotropic hormone deficiency caused by a new mutation in the TPIT gene.. Pediatrics.

[8] Adan L, Souberbielle J-C, Brauner R (1994). Management of the short stature due to pubertal delay in boys.. J Clin Endocrinol Metab.

[9] McEachern R, Drouin J, Metherell L, Huot C, Van Vliet G (2011). Severe cortisol deficiency associated with reversible growth hormone deficiency in two infants: what is the link?. J Clin Endocrinol Metab.

[10] Louvel M, Marcu M, Trivin C, Souberbielle J-C, Brauner R (2009). Diagnosis of growth hormone (GH) deficiency: comparison of pituitary stalk interruption syndrome and transient GH deficiency.. BMC Pediatr.

[11] Shulman DI, Palmert MR, Kemp SF, for the Lawson Wilkins Drug and Therapeutic Committee (2007). Adrenal insufficiency: still a cause of morbidity and death in childhood.. Pediatrics.

[12] Nasrallah MP, Arafah BM (2003). The value of dehydroepiandrosterone sulphate measurements in the assessment of adrenal function.. J Clin Endocrinol Metab.

[13] Bremer AA, Ranadive S, Conrad SC, Vallette-Kasic S, Rosenthal SM (2008). Isolated adrenocorticotropic hormone deficiency presenting as an acute neurologic emergency in a peripubertal girl.. J Pediatr Endocrinol Metab.

[14] Krude H, Biebermann H, Luck W, Horn R, Brabant G (1998). Severe early-onset obesity, adrenal insufficiency and red hair pigmentation caused by POMC mutations in humans.. Nat Genet.

[15] Jackson RS, Creemers JW, Ohagi S, Raffin-Sanson ML, Sanders L (1997). Obesity and impaired prohormone processing associated with mutations in the human prohormone convertase 1 gene.. Nat Genet.

